# Annotations

**Published:** 1899-01-21

**Authors:** 


### ANNOTATIONS.

### Isolation Hospitals.

OF the building of hospitals there seems to be no end; and if every little sanitary authority is to have its own little fever hospital and its own little small-pox hospital, we may look forward to such a multiplication of these institutions as shall bring much ridicule on our system of local self-government. The Isolation Hospitals Act is an admirable measure which gives local sanitary authorities power to combine for hospital purposes, and gives the County Council power to force them to combine to form "hospital districts," if they do not provide themselves with the accommodation which is necessary. Unfortunately, the Act gives no power to force into a "hospital district" any sanitary authority which is willing to provide for its own wants, and especially makes no arrangement for the compulsory co-operation of urban districts with the surrounding rural areas for hospital purposes. The last instance of the difficulties which arise from the independent action of local authorities is to be found at Gloucester, where a Local Government Board inquiry has just been held in regard to the erection of a new fever hospital for the city. Now Gloucester City, which contains about 40,000 inhabitants, has around it the Gloucester Rural District of between 10,000 and 15,000 inhabitants, which is managed by the Gloucester Rural District Council, and it is within the area of this rural district that the city hospital is to be erected. Not unnaturally then the Rural Council wishes to co-operate in the erection and the use of this hospital, and so save themselves from having to put up a separate one of their own. But no. There is no power to compel an authority to combine if it is willing to provide accommodation for itself, and so unless some arrangement is come to we are threatened with four little isolation hospitals—two for fever and two for small-pox—planted round one little town!

### The Club Doctors and the Price of Antitoxin.

A LARGE and somewhat serious ethical question is raised by the wide professional acceptance of the antitoxin treatment of diphtheria as *the* treatment *par excellence* of the earlier stages of that disease. When a case is removed to the hospitals of the Metropolitan Asylums Board the patient at once gets the benefit of the treatment, and whenever, from lack of beds, a patient cannot be received, a sufficient dose of the drug is put at the disposal of the practitioner by whom the case is certified for the immediate necessities of the case; certain arrangements also are made by which a supply of the serum is kept by some of the local sanitary authorities for use within their districts; but except for such provisions as these, which by no means cover the field, dependence has to be placed upon the commercial supply of antitoxin. Now all the antitoxin in the market is very dear, and when we find from the price lists of some houses that an average dose (say 6,000 units) costs thirty shillings we see how awkward is the position of the general practitioner who has to supply it. He must either deny the efficacy, and, therefore the necessity of the drug, or must incur great loss in supplying it, or must boldly admit that the fees which he accepts for the traditional "visit and medicine" do not cover, and cannot be expected to include, a supply of what is necessary for proper treatment according to the best known methods. Here is undoubtedly a serious ethical question, which goes far beyond antitoxin, and, indeed, must lead people to ask how far the cheaper type of general practitioner can for the ordinary charge supply to his patients the best treatment that is known, or even the best that he knows. Let us take the case of the club patient paying between four and five shillings a year for medicine and attendance. There are plenty of men in good practice who accept these fees; in fact, so long as the Metropolitan Provident Medical Association fixes its subscriptions at about this rate, and pays its medical officers only a portion of the money received, we can hardly expect the private clubs to rise to a much higher scale. To what extent, then, do these gentlemen supply their patients with the best known treatment for the diseases from which they suffer? The question is brought to a point by the case of antitoxin, a drug one single dose of which costs nearly as much as the doctor will receive from his patient in eight years! It is easy to say that this is reducing the matter to an absurdity. So it is; but behind the question of antitoxin are many others which the increasing cost of first-class medical and surgical treatment renders of more and more urgency every year. Many hard things are said about the action of the Friendly Societies and the members of medical clubs in beating down the remuneration given to their medical officers, and with all such criticisms we heartily agree. But something must also be said about the action of medical men in accepting fees which they must know cannot enable them to "do their best" for their patients. So long as it was merely a matter of the doctor working for small remuneration nothing need be said, for each man might fairly be allowed to assess the value of his own services. But when we find the mere cost of treatment outrunning the fees to such an extent as to entirely preclude the medical men who accept them from doing their best for their patients, it is time to draw attention to what we venture to say is a very serious ethical difficulty.

### Sewage-flooded Cellars.

IN a report to the Vestry of St. George's, Southwark, Dr. Waldo (the medical officer of health) discusses a matter which is becoming one of great urgency in all the low-lying districts of the metropolis, namely, the recurrent floodings of the cellars by sewage regurgitating from the drains. Owing to the steady and continuous increase in the extent of the area which is paved or roofed, and the imperviousness of the materials now so largely used for paving, rain runs off so quickly that even moderate storms quite fill the drains, and the low-lying houses have their cellars filled up with filth. Among the sewage-flooded cellars recently visited by Dr. Waldo were some used as work-rooms by bakers, cooks, printers, a butcher, a meat pickler, &c. One need not insist on the impropriety of cellars liable to flooding being used for any such purposes. The pity is that houses were ever allowed to be built in such low-lying positions. But there they are, and it becomes a serious question how far the sanitary authorities should permit them to be used either as dwelling-houses or for the preparation of food. Unfortunately the County Council is itself the culprit, in that its sewers are not large enough.

